# The influence of red blood cell deformability on hematocrit profiles and platelet margination

**DOI:** 10.1371/journal.pcbi.1007716

**Published:** 2020-03-12

**Authors:** Benjamin Czaja, Mario Gutierrez, Gábor Závodszky, David de Kanter, Alfons Hoekstra, Omolola Eniola-Adefeso

**Affiliations:** 1 Computational Science Lab, Faculty of Science, Institute for Informatics, University of Amsterdam, Amsterdam, Netherlands; 2 Department of Chemical Engineering, University of Michigan, Ann Arbor, Michigan, United States of America; 3 Department of Hydrodynamic Systems, Budapest University of Technology and Economics, Budapest, Hungary; North Carolina State University, UNITED STATES

## Abstract

The influence of red blood cell (RBC) deformability in whole blood on platelet margination is investigated using confocal microscopy measurements of flowing human blood and cell resolved blood flow simulations. Fluorescent platelet concentrations at the wall of a glass chamber are measured using confocal microscopy with flowing human blood containing varying healthy-to-stiff RBC fractions. A decrease is observed in the fluorescent platelet signal at the wall due to the increase of stiffened RBCs in flow, suggesting a decrease of platelet margination due to an increased fraction of stiffened RBCs present in the flow. In order to resolve the influence of stiffened RBCs on platelet concentration at the channel wall, cell-pair and bulk flow simulations are performed. For homogeneous collisions between RBC pairs, a decrease in final displacement after a collision with increasing membrane stiffness is observed. In heterogeneous collisions between healthy and stiff RBC pairs, it is found that the stiffened RBC is displaced most. The influence of RBC deformability on collisions between RBCs and platelets was found to be negligible due to their size and mass difference. For a straight vessel geometry with varying healthy-to-stiff RBC ratios, a decrease was observed in the red blood cell-free layer and platelet margination due to an increase in stiffened RBCs present in flow.

## Author summary

The primary function of the human red blood cell is to deliver oxygen to the tissues of the body. The ability of the red blood cell to deform allows it to squeeze through small capillaries along with helping transport platelets to damaged areas on the vessel wall, which initiates normal coagulation function. Many diseases are known to impede the deformability of the red blood cell, which may adversely impact the transport of red blood cells and platelets in flow. We observe that with increasing fractions of stiff red blood cells in flow, there is a decrease of platelet margination. Specifically, this is observed as a decrease of the fluorescent platelet signal at the wall of a glass channel with flowing whole blood. We confirm this margination decrease by performing cell resolved simulations that reproduce the experiments. We also observe, in simulation, a decrease in the red blood cell-free layer as red blood cell deformability decreases. The decrease in the cell-free layer, we believe, is the most significant contributing factor to decreased margination.

## Introduction

Whole blood is a complex suspension of cells, and its rheology is highly dependent on the cellular components of blood. The red blood cells (RBCs) are the most numerous cellular component, and due to their high deformability and unique bi-concave shape, they give rise to many effects of whole blood such as shear thinning [1], the Fåhraeus-Lindqvist effect [2], and the existence of a red blood cell-free layer (CFL) [3, 4]. The deformability of the RBC allows it to squeeze through the smallest capillaries of the body to successfully deliver oxygen to the tissues of the body [5].

There exist many pathologies that impair the deformability of the RBC. In sickle cell anemia, a mutation in the gene encoding for hemoglobin results in the production of hemoglobin S (HbS), which has a high tendency to polymerize [6]. HbS, located entirely in the interior of the RBC, when polymerized, creates a polymer nucleus inside the cell [7, 8]. Oxidative stress also occurs leading to cross-linking of the spectrin network resulting in a stiffened RBC membrane [9, 10]. This altered deformability of the RBCs can lead to vaso-occlusion [11] and loss of nutrients to the tissues of the body. The polymers can be long enough to cause the RBC to form a sickle shape [6]. In malaria, changes in RBC deformability occurs through plasmodium falciparum parasites [12, 13], specifically increasing the level of cross-linking of spectrin through protein–protein interactions [14]. In diabetes, RBCs undergo oxidative stress as result of being exposed to increased levels of reactive oxygen species in the body [15], resulting in reduced RBC deformability [16, 17]. Oxidative stress seems to be a common story-line from other pathologies that also report decreases in RBC deformability, such as the human immunodeficiency virus [18, 19], Parkinson’s disease [20, 21], and hereditary spherocytosis [22, 23]. Understanding the changes in the rheology of whole blood as a result of decreased RBC deformability may contribute to the understanding of these diseases.

Recent studies probing RBC deformability have found that individually stiffened RBCs marginate to the vessel walls in mice capillaries [24], as well as in glass micro-channels [25]. Microfluidic experiments found a decrease in self-organization in quasi-2D confined flows with decreasing RBC deformability [26]. *In vitro* work also found that with increasing populations of rigid RBCs in flow reduces the amount of leukocyte adhesion to P-selectin coated substrates [27] and recently to an inflamed endothelium [28]. Previous numerical studies found that the rigid body inside a malaria-infected RBC significantly affects the RBC adhesive dynamics and increases whole blood viscosity [29], as well as the reduction of contact area to the vessel wall in sickled RBCs [30].

In this work, we report on a decrease of platelet margination to the wall of a glass channel in flowing whole blood with increasing amounts of stiffened RBCs. We simulate this experiment with a cell resolved blood flow model that allows us to uncover and detail the root cause of this margination decrease for a range of rigid RBC volume fractions present in the flow. We first present *In vitro* bulk blood flow experiments. These experiments were performed by perfusing human blood with varying amounts of artificially stiffened RBCs through a 100*μm* channel and measuring flowing cell distributions via confocal fluorescent microscopy, leveraging the Z-stack capability. Cell resolved numerical simulations are then performed to confirm the results of the experiments and investigate what cannot be captured by the experiment. We use the validated cell resolved blood flow model HemoCell [31–34], which is a Lattice Boltzmann method (LBM) for the blood plasma, and a discrete element method for the RBC material model that is coupled to the plasma via the immersed boundary method. With HemoCell, we first study the influence of RBC deformability on the cell-pair level with homogeneous and heterogeneous RBC-RBC collisions, as well as collisions between platelets and RBCs. We then simulate the formation and evolution of RBC hematocrit profiles and platelet margination in bulk flow in straight vessel geometries with varying healthy/stiff RBC fractions. In both *in vitro* and numerical experiments, we observe a decrease of platelet margination, or localization, to the vessel wall with the increase in the rigid RBC population. In the simulations we also observe a decrease in CFL with increasing fractions of stiffened RBCs in flow.

## Results

### Confocal measurements of cellular distributions

Cells are stiffened by incubating RBCs with tert-butyl hydroperoxide (TBHP), inducing oxidative stress on the cell membrane. In this case, TBHP can be a general model for cell membrane perturbations resulting from oxidative disorders [35], including sickle cell anemia [10] and diabetes [36], as well as oxidative stress due to extended blood bank storage [37]. Treating the RBCs in increasing concentrations of TBHP results in increasing RBC stiffness, from moderate 0.5 mM TBHP, to moderately stiff 0.75 mM TBHP, and highly stiff 1.0 mM TBHP. We classify RBC stiffness by the elongation index (EI), Eq 1, of RBCs in a uniform shear flow environment, specifically by measuring both the minor *B* and major *A* axes.

$$\begin{array}{c} EI=\frac{A-B}{A+B} \end{array}$$ (1)

**Fig 1:**
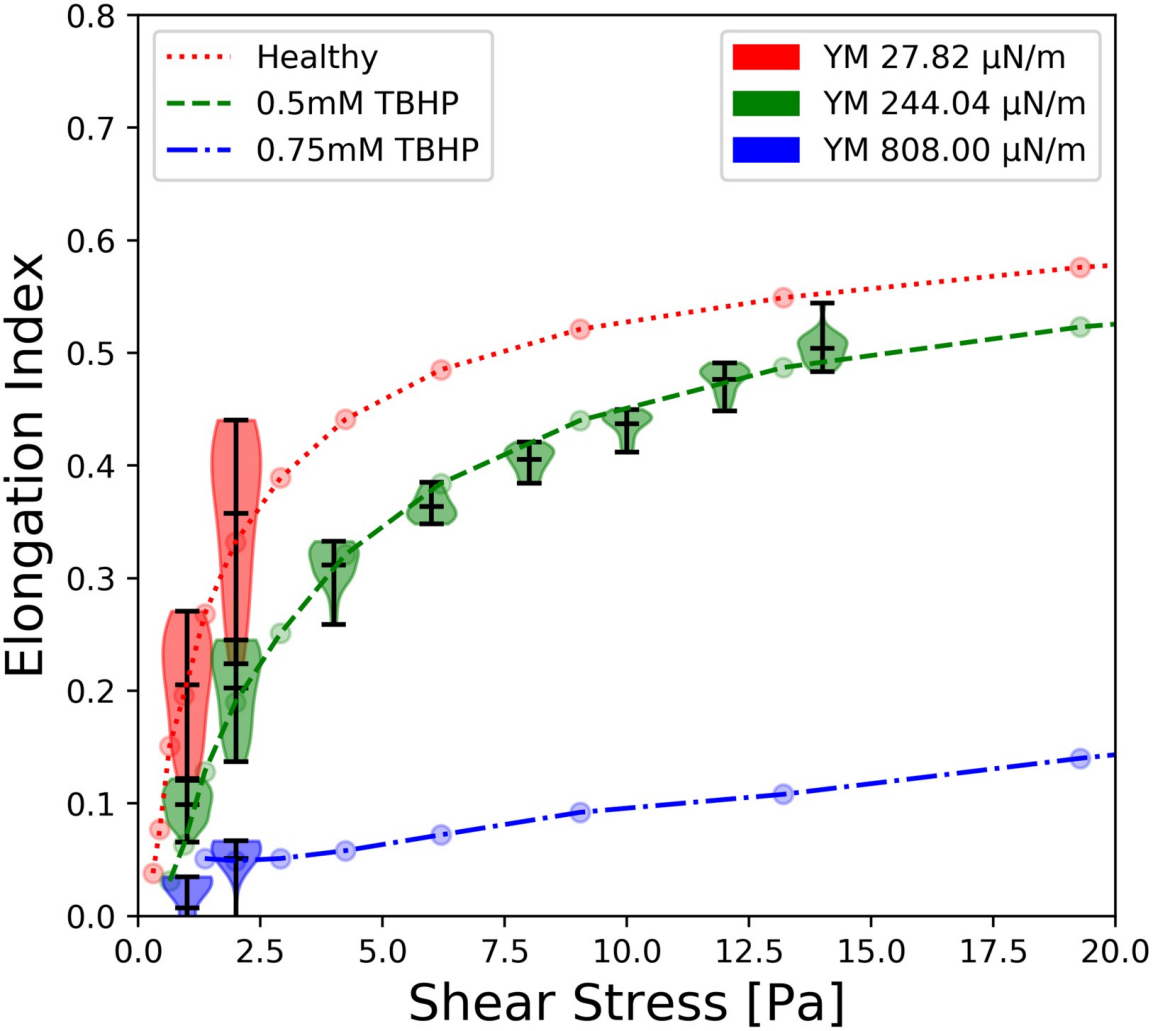
Fit of the HemoCell RBC models (violins) to the elongation curves from the ektacytometry data (dashed lines). The elongation indices from ektacytometry are colored red for healthy RBCs, green for RBCs treated in 0.5 mM TBHP, and blue for RBCs treated with 0.75 mM TBHP. Each numerical RBC is labeled by its resulting Young’s modulus (YM), and is shown as the violins which are colored red (YM 27.82 *μ*N/m), green (YM 244.04 *μ*N/m), and blue (YM 808.00 *μ*N/m) to match the corresponding experimental models. The width of each violin plot highlights the distribution of elongation indices, Eq 1, computed over a time range of 35 milliseconds. The top, middle, and bottom horizontal black bars in each violin show the location of the max, mean, and min elongation index of the numerical models respectively.

The experimental elongation index curves were obtained following the ektacytometry measurement methods from Gutierrez *et al*. [28]. Elongation indices were measured from the resulting ellipsoid laser diffraction pattern from deformed RBCs in pure shear using an ektacytometer and are shown as dashed lines in Fig 1. The details of the measurements are presented in the materials and methods section. The EI curves presented in this work are only showing the experimental RBC stiffness that a numerical model could be matched to.

**Fig 2:**
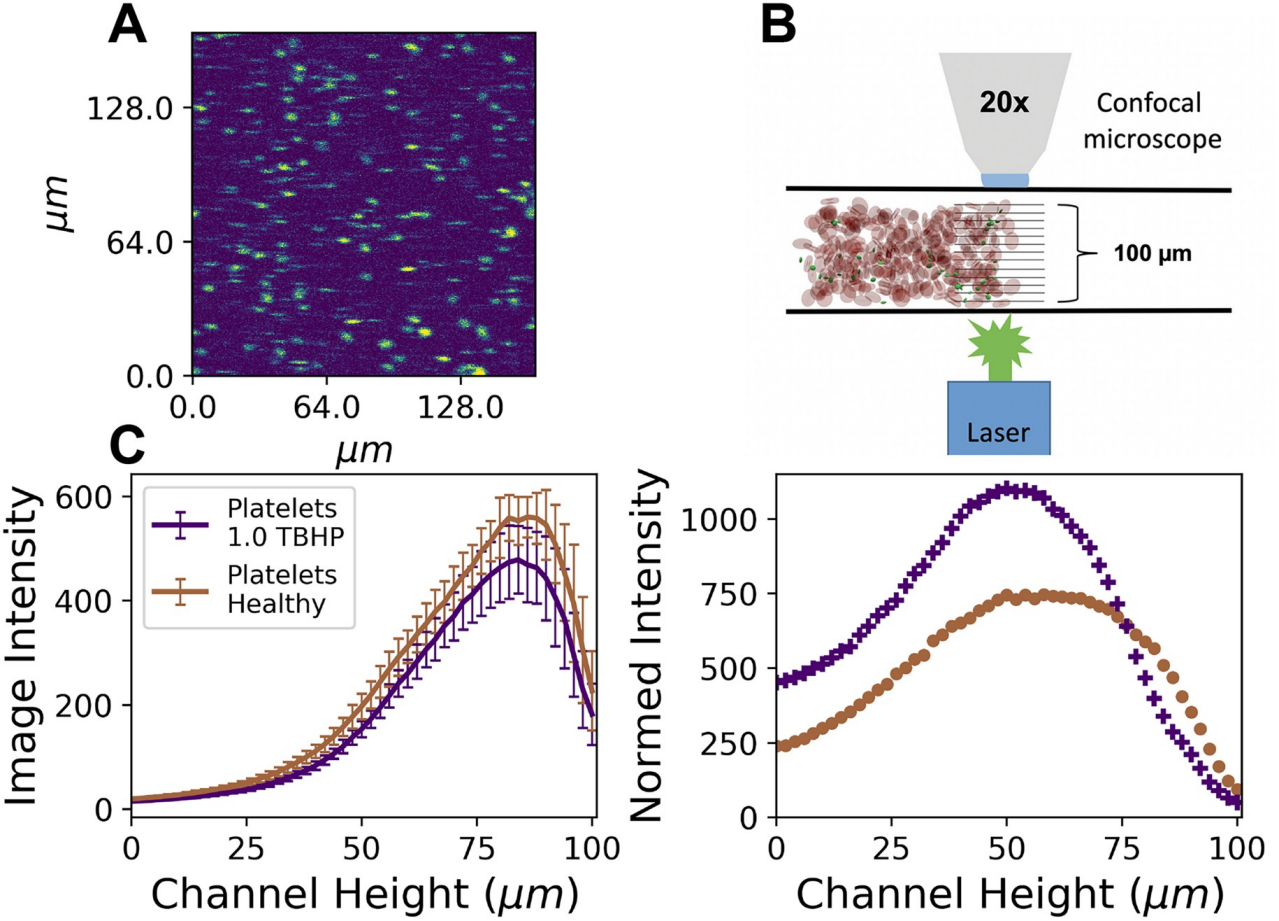
Fluorescence stained platelet confocal microscope distributions along the 100*μm* height axis of an ibidi (Gräfelfing, Germany) glass channel. 10% of platelets were stained with Allophycocyanin (APC) anti-human CD41/CD61 which was excited by a λ = 633*nm* laser and has a fluorescence emission peak at wavelength λ = 660*nm*. (A) Image of the stained platelets at the wall (channel height of 96*μm*). (B) Schematic of the experimental setup of the confocal microscope with the ibidi glass channel and the flowing blood with fluoresces stained platelets. (C) The left panel shows the raw platelet distributions (brown is measured from the 100% healthy case and the purple is the 100% 1.0mM TBHP case). (C) The right panel shows the normalized distributions corrected for absorbance per depth for both 30% hematocrit healthy blood and 30% 1.0mM TBHP treated blood.

The change in platelet margination to the vessel wall with an increase of stiffened RBCs present in the flow is directly measured by flowing human blood with varying stiffened RBC fractions through a parallel plate channel that is 100*μm* high, at a wall shear rate of 1000*s*^−1^. A small fraction of 10% of the platelets in a sample was stained with anti-human CD41/CD61 Allophycocyanin (APC) and observed via confocal fluorescent microscopy. Measurements were made by successive Z-stack images starting at the bottom of the channel, away from the objective, successively capturing images in 2*μm* height increments until reaching the top wall of the channel at 100 *μm*. The raw platelet distributions, as measured in the 100% healthy and 100% stiff case, are compared in the left panel of Fig 2C. Scans were repeated three times per measurement per individual donor. We used blood from *n* = 3 unique human donors, and error bars highlight the standard deviation of all measurements in the two panels of Fig 2C. The schematic of the experimental setup is shown in Fig 2A.

The platelet distributions are asymmetric across the channel height due to the high-volume fractions of RBCs (30% tank hematocrit) used, which causes the decay of the platelet fluorescence signal from the top of the channel (100*μ*m) near the objective to the bottom of the glass channel (0*μ*m). Given the two extreme cases, we observe that the 100% healthy case exhibits a brighter signal across the entire channel, and we attribute this to the reduced optical depth of blood containing RBCs treated with TBHP. Though TBHP induces oxidative stress on RBCs to stiffen them, it also affects the optical properties of RBCs due to the interaction of TBHP with the hemoglobin that is contained within the cell. This TBHP-hemoglobin interaction creates a more opaque suspension of blood treated with TBHP absorbing the emitted light from the fluorescently tagged platelets resulting in a dimmer platelet signal across the channel. This absorption disparity is corrected for each sample by measuring the absorbance per sample using a UV-VIS spectrophotometer. The specifics on obtaining the absorption spectra for each stiff fraction sample are elaborated in the Material and Methods section and shown in S1 Fig. Using the measured absorbance of each sample, left panel of S1 Fig, we calculate the transmittance per sample depth at the peak emission wavelength of the tagged platelets, right panel of S1 Fig, then normalize the raw platelet signals by their perspective transmittance per depth across the height of the channel. We assume that flowing blood is at a constant 30% hematocrit across the entire parallel plate channel. This assumption is a conservative overestimation, as it is known that whole blood at these scales exhibits a lower “tube” hematocrit compared to the discharge or tank hematocrit [38, 39], which is exhibited in the pipe flow simulations of this study. The normalized distributions of the 100% healthy and 100% stiff samples are shown in the right panel of Fig 2C. Error bars represent the standard deviation propagated from the multiple donors, the absorbance, and the repeated scans.

**Fig 3:**
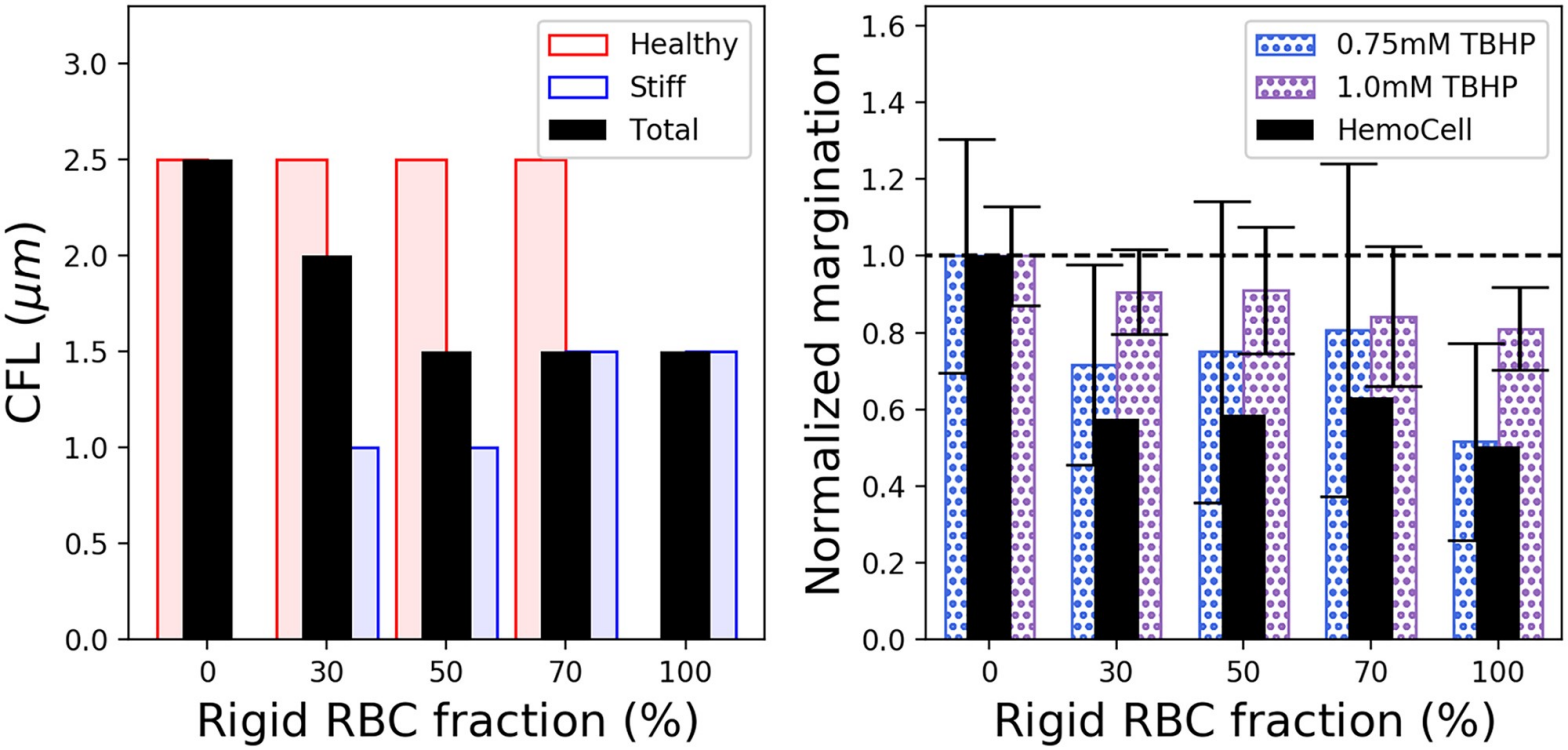
Red blood cell-free layer (left panel) and platelet margination (right panel) as a function rigid RBC fractions. The computed CFL from HemoCell is shown in black, with each of the RBC components shown in red for healthy and blue for 1.0mM TBHP. Platelet concentration at the wall is computed in the volume 4*μm* from the wall normalized to the concentration of the 100% healthy RBC case (HemoCell:black and *in vitro* results for 0.75mM TBHP:blue and 1.0mM TBHP:purple).

The platelet signal in the region 5*μm* at the closest wall to the microscope objective is averaged, and this measurement is defined as the platelet concentration at the channel wall. Each platelet concentration per stiff RBC fraction is plotted as bars in the right panel of Fig 3, normalized to the 100% healthy case. RBC samples treated with 0.75 mM TBHP are shown as blue bars, and RBCs treated with 1.0 mM TBHP are shown as purple. There exists a decrease in all experimental cases as well as the simulated cases, shown as black bars. It should be noted that a gradual decrease in platelet margination as the fraction of stiff RBCs gradually increases is not observed. The platelet signal follows the trend in CFL. The inclusion of at least 30% stiff RBCs is enough to decrease the CFL and platelet margination.

### Stiffened red blood cell numerical model

Direct simulation of the *in vitro* experimental analysis presented in this report was carried out using the cell resolved blood flow simulation software HemoCell (https://www.hemocell.eu/). HemoCell has been validated to reproduce the mechanical responses of an individual, healthy, RBC induced by sheared flow and optical tweezers, as well as accurately reproducing bulk flow hallmarks of whole blood such as the Fåhraeus-Lindqvist effect and the CFL [31]. With this validated RBC model HemoCell has been used to study the effects of RBC cytoplasmic viscosity contrasts in bulk flow [32], the role of hematocrit profiles on cell diffusivities in flow [33], as well as identifying the start of a platelet aggregate [40]. The capabilities of HemoCell allow the tracking of the individual suspended healthy RBCs, stiffened RBCs, and platelets that are present in the *in vitro* experiments reported in this study. This work proposes two stiff numerical RBC models which were achieved by scaling the mechanical parameters, in particular the link force coefficient *κ*_*link*_ and the internal viscosity ratio Λ of the original validated RBC model. The forces and their respective coefficients are elaborated in the materials and methods section of this report.

It was found that by increasing the link force, which captures the stretching and compression of the underlying spectrin-network, contributed most to the overall stiffening of the RBC. The link force may also be the most physiologically relevant numerical parameter to increase in order to create a stiffened RBC model, because inducing oxidative stress through TBHP treatment causes spectrin and ankyrin degradation [41]. The stiff RBC parameters are reported in Table 1, along with the original validated [31] healthy model. A surface Young’s modulus is computed for each stiffened RBC by deforming a single hexagonal patch of the membrane following the validation methods of the original model [31]. A Young’s modulus of 244.04 *μ*N/m is obtained for the 0.5mM TBHP numerical model and 808.00 *μ*N/m for the 0.75mM TBHP numerical model. It should be noted that these may not be unique combinations of scaled parameters to produce the stiffened RBCs. Numerical RBC rigidity can be achieved through scaling any of the forces that make up the HemoCell model. The smallest changes to the original validated model were chosen in order to minimize numerical instability.

**Table 1:** HemoCell model parameters.

	Link coefficient (*κ*_*link*_)	Internal viscosity ratio (Λ)	Young’s modulus (YM)
**Healthy** [31]	15 *k*_*B*_*T*	1	27.82 *μ*N/m
**0.5mM TBHP**	90 *k*_*B*_*T*	6	244.04 *μ*N/m
**0.75mM TBHP**	600 *k*_*B*_*T*	1	808.00 *μ*N/m
**Platelet** [40]	25 *k*_*B*_*T*	1	70.6 *μ*N/m

The elongation indexes were computed by placing a single RBC in a uniform sheared environment. Uniform shear was implemented by applying constant velocity + *V*_*x*_ at the top boundary + *Y* and −*V*_*x*_ at the lower boundary −*Y*. The elongation index was calculated by fitting an ellipse to the projection of the membrane after the RBC reached an equilibrium deformed state, following the procedures from a previous study [32]. Elongation indexes were averaged from 10 time points after it equilibrated, which typically occurred after a strain of *γt* = 5. The results of the matching stiffened RBC HemoCell models are scattered in the violin plots across the ektacytometry data in Fig 1.

The physiological value for the internal viscosity of a healthy RBC has been found to be between 3-10 mPas [32, 42]. Given the plasma viscosity range of 1.1-1.3 mPas [43], this leads to an approximate interior viscosity ratio range of 2-9. It was found that the increased viscosity ratio Λ for the 0.5mM TBHP cell contributed to the flattening out of the elongation index curves at stresses ≥ 5 Pa. The viscosity ratio, however, does not significantly influence cell deformability for the 0.75mM TBHP cell, as the greatly increased link force is the dominant parameter for rigidity. We therefore keep Λ = 1 to optimize computational performance, as identifying and modifying the viscosity of inner and outer LBM fluid nodes lying inside and outside an RBC is computationally taxing [32]. The 0.75mM TBHP numerical model could not sustain shear stresses above 2 Pa, or shear rates *γ* ≥ 2000*s*^−1^. Therefore only the stable models are reported, which are relevant to the cell-pair collision simulations, and the straight vessel simulations, where shear rates do not exceed 1200*s*^−1^. For the remainder of this report, RBC stiffness is referred by the amount of TBHP for both *in vitro* and *in silico*. Only the 0.75mM numerical model is employed in the bulk flow simulations. Matching a numerical model to the 1.0 mM TBHP ektacytometry data could not be achieved as the numerical model became unstable at shear stresses ≥ 2.0 Pa.

### Cell pair collisions

**Fig 4:**
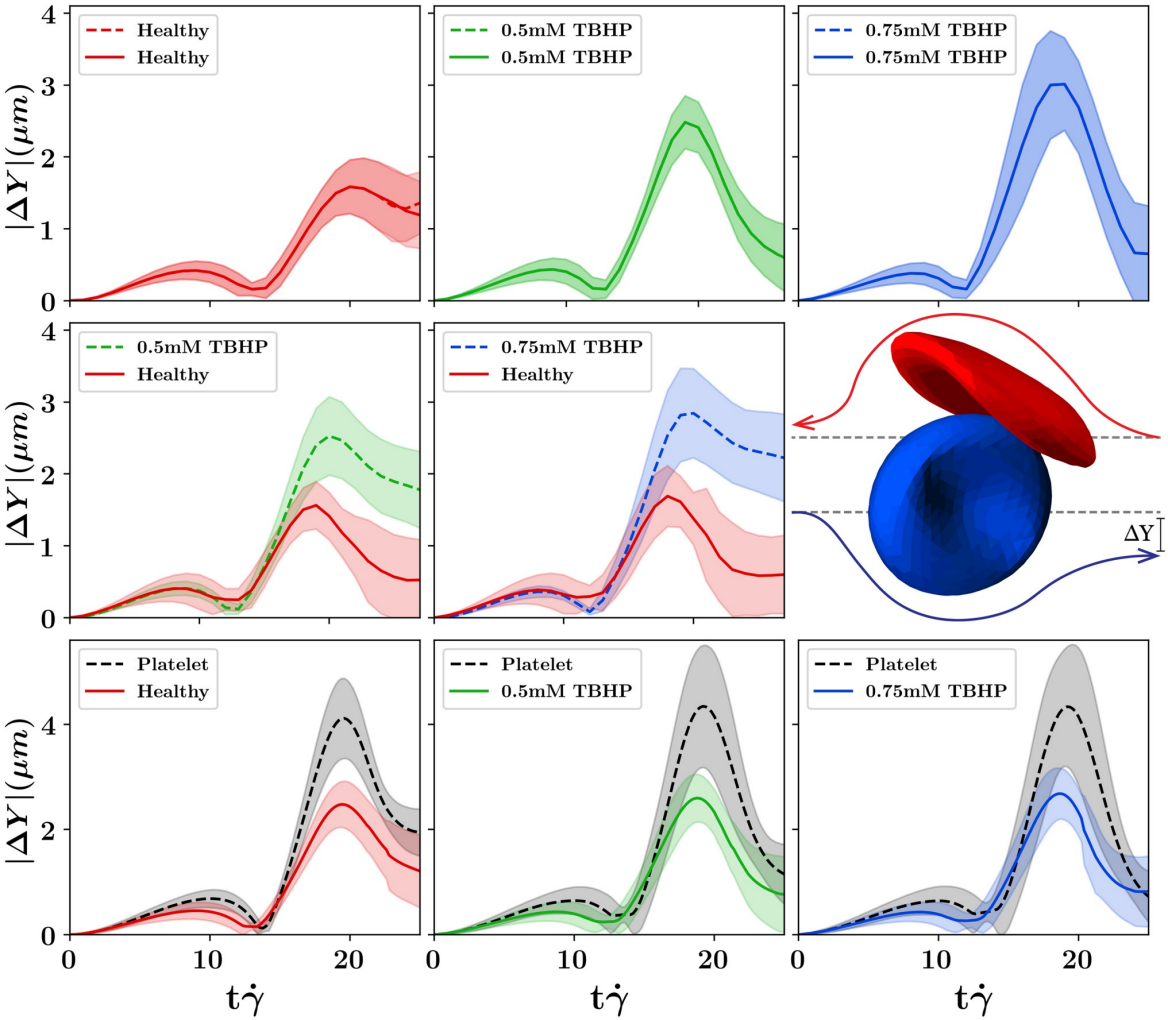
Displacement trajectories per strain from collisions between single RBCs of varying membrane stiffness. The top row is homogeneous RBC collisions between alike stiffness pairs, the middle row shows heterogeneous RBC collisions between a healthy RBC and a stiff RBC, and the bottom row shows collisions between RBCs and platelets. The color scheme throughout the figure is red for healthy RBCs, green for 0.5 mM TBHP RBCs, blue for 0.75 mM TBHP RBCs, and black for platelets. The schematic of the cell collisions and computed displacement |Δ*Y*| is shown in the right panel of the middle row. The spread of each line highlights the standard deviation of the trajectories resulting from different orientations of initial RBC positions.

First, the effect of RBC deformability on the cell-pair level by simulating collisions between RBC pairs of varying stiffness was probed. In this simulation setup, two cells are placed at a distance of *X* = 16*μm* apart, and a uniform shear is applied to the LBM fluid by setting a constant velocity *V*_*x*_ and −*V*_*x*_ at the top and bottom (*Y*) boundaries of the simulation. The cells are offset by a distance of *δY* = 2*μm* in the velocity gradient direction causing the cells to collide with each other. The magnitude of the lateral displacement of each cell trajectory, with respect to the respective initial positions, was tracked. This is in the velocity gradient (*Y*) direction and reported over the characteristic time of a single collision, i.e., strain, shown in Fig 4. Each pair collision was simulated 10 times, with five different initial orientations of one RBC in the X-Y plane rotating around the Z-axis (0, 22, 45, 67, and 90 degrees from the +X-axis). The initial positions of the two cells were also swapped, resulting in an additional five simulations of the same cell-pair, which was done to explore the effect of various incoming cell orientations. The mean of all trajectories per collision pair is shown as dark lines in Fig 4, and the standard deviation is shown as the shaded region per collision pair. Each collision was simulated for shear rates of 200, 500, and 1000*s*^−1^. Video of the cell collisions for each pair combination are presented in the supplementary material, S1 Video. Videos highlighting the cell trajectories given variations of the cell initial orientations is shown in S2 Video.

Homogeneous collisions (top row of Fig 4) between alike RBC pairs show that final displacements are largest when they are most deformable. In the healthy-healthy RBC collision, both RBCs can deform, more than the stiffened RBCs, during the collision, which helps them maintain closer to their original position throughout the collision. However, during the collision, some energy is lost to membrane deformation, limiting the return to their original position. This results in a smaller displacement at the peak of the collision but a larger final displacement, shown in the left panel of Fig 4. In the stiff-stiff RBC collision, cells are displaced more during the collision as they cannot deform. However, these cells will return closer to their initial positions following the collision as there is less energy loss to the deformation of the membrane. That is, for the stiff-stiff RBC collision, there is a higher maximum displacement during the collision but a smaller final displacement, shown in the middle and right panels of the top row of Fig 4. As RBC stiffness increases, the homogeneous collisions approach the time-reversible stokes regime of two colliding rigid spheres, where the stiffened RBCs exhibit less displacement after a collision.

In the heterogeneous collisions between a healthy and stiff RBC (middle row of Fig 4), the deformability of the healthy RBC allows the healthy RBC to absorb the energy of the collision maintaining closer to its original position. The stiffened RBCs cannot deform and as a result, are displaced most in the heterogeneous collision. The final displacement of the stiffened RBC increases with increasing RBC stiffness in the heterogeneous (healthy-stiff) RBC collisions. A similar effect was previously observed in heterogeneous collisions between elastic capsules [44].

The platelet-RBC cell pair collisions with RBCs of increasing membrane stiffness are shown in the bottom three panels of Fig 4. The maximum platelet displacement slightly increases with RBC stiffness, and final platelet displacement decreases with RBC stiffness. Following the same trend as the homogeneous RBC collisions which approach the time-reversible stokes regime as RBC stiffness increases. It should be noted that this effect is relatively small, and the platelet-RBC collision is mostly influenced by the disparity of size between the two [33]. Although there is a small effect of RBC stiffness with platelet displacement, RBC deformation is mostly negligible in any RBC stiffness scenario as the RBC is much larger, which contributes most to the displacement of the platelet.

### Bulk rheology including stiff red blood cells

To study the behavior of stiffened RBCs in bulk rheology, we simulate whole blood flowing through a periodic pipe of radius *R* = 50*μm* with a tank hematocrit of 30% driven by a body force resulting in a wall shear rate of 1000*s*^−1^. We varied the fraction of stiff/healthy RBCs (0/100, 30/70, 50/50, 70/30, and 100/0) in each simulation, maintaining a total hematocrit of 30%. All of the bulk pipe flow simulations used the numerical 0.75mM TBHP stiff RBC model, as a stable numerical model of the 1.0mM TBHP treated cell could not be obtained. Due to the computational cost of the simulations, the stiffer 0.75mM TBHP model was chosen over the 0.5 mM TBHP model in order to better highlight the differences between stiffened and deformable RBCs in flow. Videos of the 50/50 mixture are also included, S3 Video, showing the bulk flow simulation, a cross-section of the cells in flow, and the radial volume fraction profiles of cells over time.

#### Red blood cell radial diffusivity and hematocrit profile

**Fig 5:**
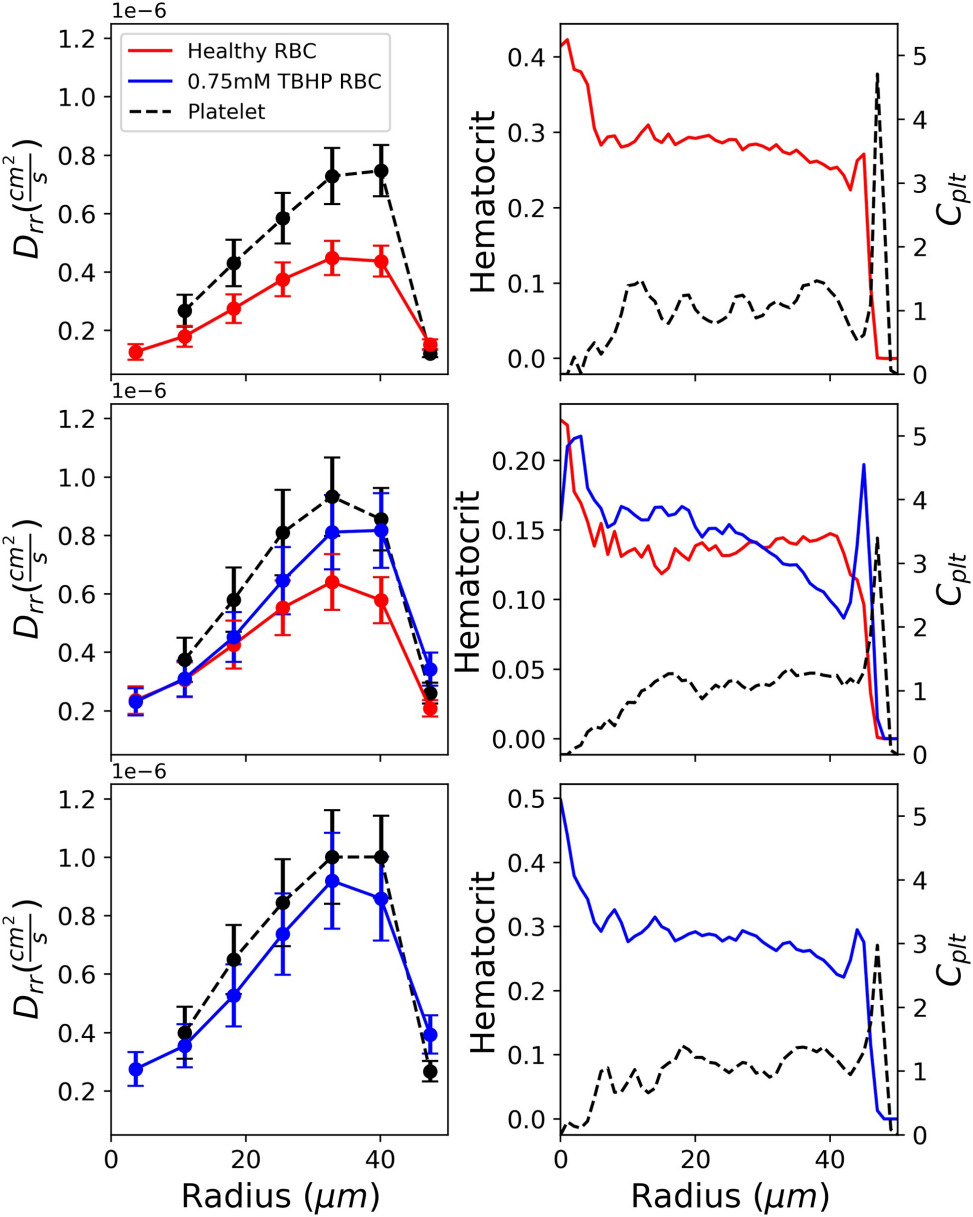
Simulation results of diffusion coefficient profiles (left panels) and volume fractions (right panels) along the radial axis of a 50*μm* radius pipe with a tube hematocrit of 30%. The top row is the 100% healthy case, the middle row is the 50/50 healthy/stiff case, and the bottom row is the 100% stiff case. The right panels show volume fraction profiles across the radius of the pipe for healthy RBCs:red, 0.75mM TBHP RBCs:blue, and platelets:black. *C_plt_* is the platelet volume fraction, which is defined as the ratio of the local platelet volume fraction divided by the mean vessel platelet volume fraction.

The radial diffusion coefficient *D*_*rr*_ of each RBC type was computed in seven radial regions in the straight vessel via tracking cell trajectories in each of the radial regions. The radial regions are centered at (3.6, 10.9, 18.2, 25.5, 32.8, 40.0, and 47.4)*μ*m and each being approximately 7.3*μ*m wide. The diffusion coefficients were computed per radius region and averaged over 0.4 seconds, with a time window of 0.5ms. We follow the specific procedure from a previous study [33] and confirm that RBC radial diffusion increases from the center of the pipe towards the wall, following the increase of shear rate. Radial diffusivities are shown in the left panels of Fig 5. With stiffened RBCs in the simulations, an increase of RBC *D*_*rr*_ in all regions of the pipe is observed. The 100% healthy case shows the lowest RBC *D*_*rr*_, which we attribute to deformable RBCs exhibiting the smallest maximum displacement during the single cell homogeneous collisions. The 100% stiff simulation, however, exhibits the highest RBC *D*_*rr*_ due to the largest maximum displacement during collision observed in the homogeneous collisions. In the 50% healthy 50% stiff mixture, we observe higher RBCs *D*_*rr*_ for both healthy and stiff RBCs compared to the healthy case. The stiff RBCs also exhibit larger diffusivities compared to the healthy RBCs in this mixture which is influenced by the heterogeneous collisions. The heterogeneous stiff-healthy RBC collisions result in a larger final displacement of the stiffened RBC. Therefore in the high shear rate radial region (25*μm* ≤ *R* ≤ 45*μm*) a higher stiff RBC diffusion coefficient is observed, resulting in a clearing out of stiff RBCs from this region by healthy RBCs as shown in Fig 5 (middle right panel). The clearing out of stiffened RBCs from this region results in a stiff hematocrit peak at the vessel wall and more stiff RBCs towards the center of the channel.

The RBC free layer decreases as a result of an increase of stiffened RBC fraction in flowing blood, left panel of Fig 3. In general healthy RBCs exhibit a larger CFL(red bars in the left panel of Fig 3) compared to the stiff RBCs (blue bars in the left panel of Fig 3), which is consistent across all stiff/healthy RBC mixtures. Since stiff RBCs do not deform easily under shear and maintain their bi-concave shape, we expect that the decrease of the CFL is partly caused by the absence of tank-treading of the stiff RBCs close to the vessel wall. Tank-treading motion at the vessel wall has been shown to influence the transverse drift of vesicles in a sheared suspension [45, 46], which promotes the lift force felt by RBCs at the vessel wall creating a CFL [47].

#### Platelet radial diffusivity and volume fraction profile

The radial diffusion coefficient *D*_*rr*_ of the platelets was also computed in the seven radius regions in the straight vessel. We observe an increase in platelet diffusivities in each radius region, as the fraction of stiffened RBCs in flow increases. Platelet radial diffusion coefficients are shown in left panels of Fig 5. Platelets do exhibit a larger maximum displacement when RBC stiffness increases. However, previously observed in the platelet-RBC pair collisions, the maximum platelet displacement is largely dictated by the mass difference of the two cells and not significantly affected by changing RBC stiffness. Since RBCs, both healthy and stiff, are being displaced more, exhibiting larger diffusivities due to the presence of stiffened RBCs, platelets are also affected as they are also colliding with the RBCs. Therefore, the increase of platelet diffusivities could be a secondary result of the increase of RBC diffusivities as the fraction of stiffened RBCs present in flow increases.

A decrease of platelet localization at the vessel wall as the fraction of stiffened RBCs increase is also observed, as highlighted in the platelet volume fraction profiles shown in the right panels of Fig 5. Here, platelet volume fraction *C*_*plt*_ is reported as the fraction of the local radial platelet volume fraction $C_{plt}^{*}$ to the mean vessel platelet volume fraction $\bar{C_{plt}}$. Platelet concentrations in a 5*μ*m wide volume at the wall are directly measured and shown normalized to the 100% healthy case in the right panel of Fig 3. We observe a step decrease in platelet concentration at the vessel wall when stiffened RBCs are introduced into the flow, even in the 30% stiff case.

### Conclusions

In this research, we provide *in vitro* and *in silico* evidence on the direct impact of rigid RBCs in flowing blood. Previous research addressing this issue largely focuses on the effect of stiffened RBCs present in small fractions in the blood [24, 25, 29, 30], while diseases characterized by stiff RBCs have a range of fractions from patient to patient. We investigate the influence of RBC deformability when a significant percentage of stiffened RBCs are present in whole blood. We measure a decrease in the localization of platelets at the channel wall *in vitro*. Due to the high hematocrits considered, the platelet fluorescence signal is considerably attenuated in our experiments, limiting the depth of which we can resolve the full-flowing distribution of platelets. We believe that this will be a general limitation when trying to observe cell distributions in flowing whole blood of hematocrits ≥ 30%. We were able to simulate this experiment with the blood flow model HemoCell allowing us to resolve what we could not see in our experiment.

Recent microfluidic work reported that with an increase of stiffened RBCs present in flow, stiffened RBCs concentrate more toward the center-line of a 50*μ*m wide channel [48], when compared to a healthy case. We do not observe a significant difference in stiff vs. healthy hematocrit profiles. Given the vessel diameters considered in this study are much as larger, we believe this effect becomes more significant for vessels with diameters ≤ 50*μ*m.

Numerical works have hinted at a decrease in platelet margination with an increase of stiffened RBCs [49, 50] as a result of diabetes, speculating that this may be caused by a decrease in collisions between RBCs and platelets when only stiffened RBCs are present in the flow. We, however, find that increasing the amount of stiffened RBCs in flow causes an increase in the number of collisions, both heterogeneous and homogeneous, as observed with higher diffusion coefficients, for both platelets and RBCs, corresponding to higher amounts of stiffened RBCs present.

Platelet margination, we believe, is then likely altered by the decrease in size of the CFL as there is limited volume next to the wall for platelets to be trapped in, highlighted in the left panel of Fig 3. The decrease in size of the CFL could be attributed to a lessened wall lift force felt by the stiffened RBCs, primarily due to the absence of tank treading motion stiff RBCs experience at the vessel wall. This could also be a contributing factor in the increase of apparent blood viscosity observed in simulation [29] and clinical blood tests [51] of malaria-infected blood. The work presented here proposes a general model for stiffened RBCs as a result of disease, both experimental and computational, and offers evidence of the detrimental effect rigid RBCs have on physiological blood flow and the ability of platelets to localize to the vessel wall.

## Materials and methods

### Study approvals and preparation of human blood

Protocols similar to the ones presented by Gutierez *et al* [28]. are applied in this work. Fresh human blood is obtained from consenting donors via venipuncture. Blood draw protocols have been approved by the University of Michigan Internal Review Board (IRB-MED). Acid citrate dextrose (ACD) was utilized as an anticoagulant. RBCs were separated from whole blood via a series of *slow* centrifugation separation steps and were thoroughly washed with phosphate buffer solution (PBS (-/-)). Platelet-rich plasma is incubated with anti-human CD41/CD61 Allophycocyanin at 37°C for 1 hour. Platelets are stained independently and are then reconstituted into whole blood. Whole blood is maintained at the desired hematocrit of 30%.

### Red blood cell rigidification

Similar to the method used by Gutierrez *et al*. [28], isolated RBCs were incubated (2% hematocrit) for 30 minutes in a solution of PBS (-/-) with a specific concentration of TBHP (0.5 mM, 0.75 mM, & 1.0 mM). After the incubation period, stiffened RBCs are thoroughly washed with PBS (-/-).

### Ektacytometry

Ektacytometry measurements of elongation index from healthy and TBHP treated RBCs were made using a laser-assisted optical rotational cell analyzer (LORRCA; Mechatronics, Hoorn, The Netherlands) [52]. Diffraction patterns are a function of increasing shear stress applied to the collection of RBCs. EI is a function of the difference between the major and minor axis of a RBC diffraction pattern over the major and minor axes sum, given in Eq 1. Blood samples were collected using the anticoagulant Ethylenediaminetetraacetic acid (EDTA), which has been shown to minimize the influence on hemorheological parameters [53].

### Confocal distribution experiments

Blood was perfused through a parallel plate glass channel of dimensions LxWxH (48.2*x*5.0*x*0.1)mm by controlling the volumetric flow rate using a syringe pump (SPLG100 Series). A 10mL syringe was pulled at a volumetric flow rate *Q* of 500*μL*/min to ensure a wall shear rate inside the channel of 1000*s*^−1^. This was computed using the wall shear stress relationship $\tau_{w}=\mu \dot{\gamma }=\frac{6\mu Q}{a^{2}b}$, where *b* = 5mm, *a* = 0.1mm, and the apparent viscosity of suspending media, *H*_2_*O* @ 37°C, *μ* = 0.00076Pa⋅s. The parallel plate flow chamber was constructed using a *μ*-Slide Luer sticky underside (ibidi) and a plain glass slide. Fluorescently tagged platelets were excited by a λ = 633*nm* laser and observed at the peak emission wavelength λ = 660*nm*. Confocal distribution measurements were made using Z-Stack images generated using a FluoView FV1000 Series Laser Scanning Confocal Microscopes from Olympus. An Olympus UMPLFLN 20XW water immersion objective with ×20 magnification and a numerical aperture of 0.5 was used with a field of view of 512x512 pixels (resolution of 0.32 microns per pixel).

### Absorbance spectra

The absorbance spectra from 28 trials per stiff fraction per TBHP treatment with independently stained platelets were loaded onto multiple 96-well plates. Each well contained a total volume of 100mL of reconstituted whole blood with 30% tank hematocrit, which corresponds to a sample path length of 3.108 mm. The plates were measured using a Synergy H1—BioTek microplate reader. The absorbance spectra was analyzed over a range of wavelengths from 600 to 700nm, including the peak emission of the platelet fluorescence λ = 660nm. The average measured absorbance per treatment and stiff volume fraction is shown in S1 Fig.

### HemoCell model and simulations

The high performance library HemoCell (High pErformance MicrOscopic CELlular Libary) was used to perform all of the simulations presented in this report. HemoCell implements a validated mechanical model of an RBC which is then capable of reproducing the emergent transport phenomena and non-Newtonian characteristics of a cellular suspension system [31]. The HemoCell RBC force model is a superposition of four discrete forces that model the stretching and compression of the underlying spectrin-network (*F*_*link*_), the bending response of the membrane arising from the non-zero thickness of the spectrin-network (*F*_*bend*_), the combined surface response of the supporting spectrin-network and the lipid bilayer of the membrane to stretching and compression (*F*_*area*_), and a force to maintain the quasi-incompressibility of the cell (*F*_*volume*_). The RBC model has been further developed to include the viscosity contrast between the internal fluid of RBCs and the surrounding blood plasma [32]. In this report only the link force and internal viscosity ratio Λ were tuned to build two new stiffened RBC models. The link force *F*_*link*_ is shown in Eq 2.

$$\begin{array}{c} F_{link}=-\frac{\kappa_{l}dL}{p}[1+\frac{1}{\tau_{l}^{2}-dL^{2}}] \end{array}$$ (2)

Here *κ*_*l*_ is the link force coefficient, $dL=\frac{L_{i}-L_{0}}{L_{0}}$ is the normal strain defined as the relative deviation from the equilibrium length *L*_0_. *τ*_*l*_ is the relative expansion ratio, chosen to be 3, when the spectrin network reaches its persistence-length. The persistence-length of a spectrin filament was chosen to be *p* = 7.5nm. The link force coefficient *κ*_*l*_ is a free parameter and is used to scale the magnitude of the link force to match experimental data.

The parameters of the HemoCell model were kept the same throughout all single cell shear flow, cell pair collision, and straight vessel bulk flow simulations. The kinematic viscosity and density of the LBM plasma was chosen as *η* = 1.1*e*^−6^*m*^2^*s*^−1^ and *ρ* = 1025*kgm*^−3^ which allows us to convert from applied shear rate to shear stress i.e. $\tau =\dot{\gamma }\eta \rho$. The LBM grid resolution was chosen to have a *dx* = 0.5*μ*m and a relaxation parameter of *τ* = 1.1 which resulted in a timestep of *dt* = 0.1*μ*s. Initial cell positions populating a tank hematocrit of 30% in the pipe flow simulation was achieved following the procedures for a kinetic process for hard ellipsoid packing [54, 55].

## Supporting information

S1 Fig Absorbance, absorbance spectra, and transmittance per sample depth. Absorbance spectra and transmittance per sample depth of 100% healthy and 100% 1.0mM TBHP treated blood, shown in the bottom two panels. The bottom left panel is the Absorbance spectra measured from a UV/VIS spectrophotometer for both 100% healthy and 100% 1.0mM TBHP treated RBCs, both samples have a discharge hematocrit of 30%. The vertical grey bar at wavelength of λ = 660*nm* highlights the peak emission wavelength of the fluorescence used to stain the platelets. The bottom right panel shows the % transmittance per sample depth at the peak emission wavelength λ = 660*nm*. The top two panels show the average measured absorbance in 30% hematocrit (tank) blood with increasing amounts of treated RBCs, for both 1.0mM TBHP and 0.75mM TBHP treatments.
(TIF)

S1 Video Simulations of cell collisions by cell type. This video shows the paths of both cells during the cell pair collisions at a shear rate of 500*s*^−1^. RBC-RBC homogeneous collisions are shown on the top row, RBC-RBC heterogeneous collisions are shown in the middle row, and platelet-RBC collisions are shown in the bottom row. The initial orientation of the incoming (left) cell face is parallel to the x-z plane at a rotation of 0 degrees from the +X axis.
(MP4)

S2 Video Simulations of cell collisions by initial cell orientation. This video shows the paths of both cells during the RBC-RBC heterogeneous pair collisions, at a shear rate of 500*s*^−1^. The initial orientation of the incoming (left) cell face is parallel to the x-z plane is varied from a rotation of 0 (left panels), 45 (middle panels), and 90 (right panels) degrees from the +X axis. The bottom row shows the collision trajectories when the two cell initial positions of the top row are swapped.
(MP4)

S3 Video Simulation of straight vessel with 50% Healthy 50% stiff RBC mixture. In this video you see the 0.55s of the 50/50 healthy stiff RBC straight vessel simulation with the evolution of each cell type volume fraction profiles along with a cross section of the straight vessel.
(MP4)
