# Supplementary figures and images for: The influence of red blood cell deformability on hematocrit profiles and platelet margination
Source: PLoS Comput Biol. 2020 Mar 12;16(3):e1007716. doi: 10.1371/journal.pcbi.1007716 (PMC7093031; doi:10.1371/journal.pcbi.1007716)

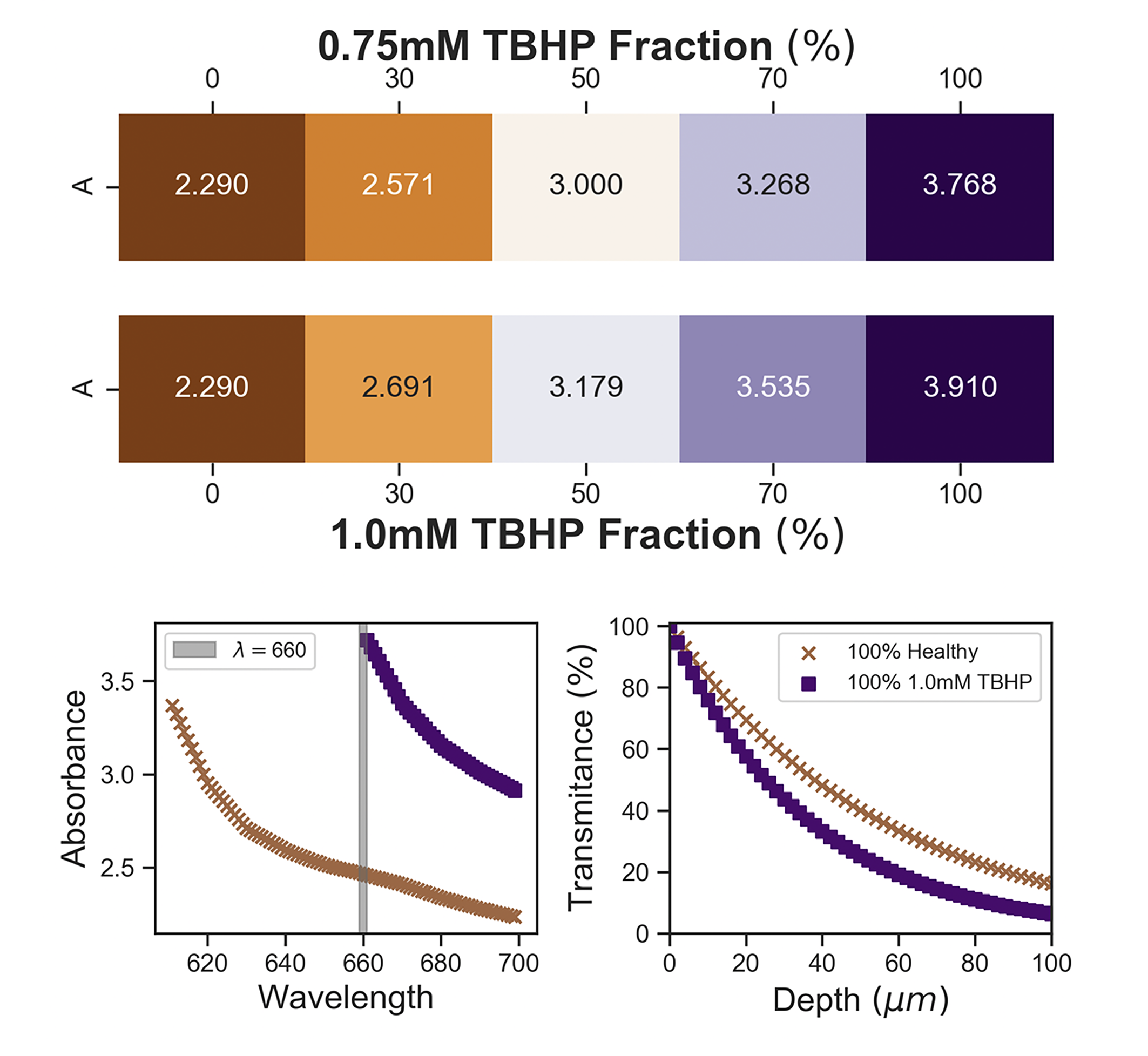

Supplement: S1 Fig — Absorbance spectra and transmittance per sample depth of 100% healthy and 100% 1.0mM TBHP treated blood, shown in the bottom two panels. The bottom left panel is the Absorbance spectra measured from a UV/VIS spectrophotometer for both 100% healthy and 100% 1.0mM TBHP treated RBCs, both samples have a discharge hematocrit of 30%. The vertical grey bar at wavelength of λ = 660nm highlights the peak emission wavelength of the fluorescence used to stain the platelets. The bottom right panel shows the % transmittance per sample depth at the peak emission wavelength λ = 660nm. The top two panels show the average measured absorbance in 30% hematocrit (tank) blood with increasing amounts of treated RBCs, for both 1.0mM TBHP and 0.75mM TBHP treatments. (TIF) [file pcbi.1007716.s001.tif]
